# Study of Viscoelastic Properties of Graphene Foams Using Dynamic Mechanical Analysis and Coarse-Grained Molecular Dynamics Simulations

**DOI:** 10.3390/ma16062457

**Published:** 2023-03-20

**Authors:** Shenggui Liu, Mindong Lyu, Cheng Yang, Minqiang Jiang, Chao Wang

**Affiliations:** 1 School of Mechanics and Civil Engineering, China University of Mining and Technology, Beijing 100083, China; 2 The State Key Laboratory of Nonlinear Mechanics (LNM), Institute of Mechanics, Chinese Academy of Sciences, Beijing 100190, China; 3 School of Engineering Science, University of Chinese Academy of Sciences, Beijing 100049, China

**Keywords:** graphene-foam materials, viscoelastic properties, dynamic mechanical analysis, microstructure, coarse-grained molecular dynamics simulation

## Abstract

As a promising nano-porous material for energy dissipation, the viscoelastic properties of three-dimensional (3D) graphene foams (GrFs) are investigated by combining a dynamic mechanical analysis (DMA) and coarse-grained molecular dynamic (CGMD) simulations. The effects of the different factors, such as the density of the GrFs, temperature, loading frequency, oscillatory amplitude, the pre-strain on the storage and loss modulus of the GrFs as well as the micro-mechanical mechanisms are mainly focused upon. Not only the storage modulus but also the loss modulus are found to be independent of the temperature and the frequency. The storage modulus can be weakened slightly by bond-breaking with an increasing loading amplitude. Furthermore, the tensile/compressive pre-strain and density of the GrFs can be used to effectively tune the viscoelastic properties of the GrFs. These results should be helpful not only for understanding the mechanical mechanism of GrFs but also for optimal designs of advanced damping materials.

## 1. Introduction

Graphene foam (GrF), a new kind of polymer material assembled by plenty of graphene sheets, has remarkable mechanical properties [1,2,3] and superior electrical conductivity combined [4,5], and shows potential applications in sensing [6], advanced composite materials [7], stretchable electronic devices [4] and energy-storage components [8].

For conventional polymer materials or their composites, viscoelastic properties measured by the storage/loss modulus and the damping ratio are highly sensitive to temperature and loading frequencies; they vary by several orders of magnitude with a change in temperature and loading frequency, e.g., the storage modulus of the graphene oxide filled polyurethane nanocomposites [9] increases from 10^3^ MPa to more than 10^4^ MPa with an increased loading frequency, and its storage modulus decreases from 400 to 10 MPa with an increased temperature. The dynamic viscosity of the graphene-polymer nanocomposites [10] decreases from 10^6^ to 10^2^ Pas with an increased frequency. It has been explained by friction and movement between the polymer chains [9,10]. In contrast, it has been found that the viscoelastic properties of GrFs are almost independent of temperature and loading frequency and the change is less than one order of magnitude. For example, Luo et al. [1] and Wu et al. [3] experimentally showed that the storage/loss modulus and the damping ratio of the synthesized three-dimensional graphene foams are insensitive to temperature in the range of −50 to 300 °C and to loading frequencies in the range of 1 to 20 Hz. Xu et al. [11] experimentally showed that the storage/loss modulus of the self-assembled graphene hydrogels are independent of the angular frequencies from 1 to 100 rad/s. Sun et al. [2] found that the assembled carbon aerogels had a stable storage/loss modulus as the temperature varies in a range of −200 to 300 °C. The temperature and loading frequencies adopted in the reported experiments [1,2,3,11] are in a limited range. The viscoelastic properties of the GrFs under the condition of temperatures higher than 300 °C and loading frequencies higher than 20 Hz has not been reported. The identification of the mechanism of the temperature- and frequency-independent viscoelastic properties is lacking up to now. Furthermore, other influencing factors, such as the density of the GrFs, the layer number of the constituent graphene sheets, the loading amplitude and pre-strain, which are always encountered in practical applications and have great influence on both the stiffness and strength of GrFs [3,12,13]. However, the effects of these factors on the viscoelastic properties of graphene foams have not been studied up to now.

The coarse-grained molecular dynamic method has been widely used in the studies of graphene foams [14,15,16,17] in recent years to analyze quantitatively the microstructure and energy distribution of graphene foam. In this paper, we adopt both the experimental dynamic mechanical analysis (DMA) method and the numerical coarse-grained molecular dynamic simulation (CGMD) to systematically study the viscoelastic behaviors of GrFs under temperature, frequency, amplitude, pre-strain and density of the graphene foam. The outline of this paper is as follows. Details on the experimental materials and the DMA instrument, the numerical model proposed by Cranford et al. [14] and the DMA methodology are given in Section 2. Section 3 shows the influence of the density temperature, frequency, amplitude and pre-strain on viscoelastic properties on the graphene foam. Finally, the conclusion is given.

## 2. Experimental Materials and DMA Instrument, Numerical Model and Methodology

### 2.1. Experimental Materials

Ultralight GrF aerogels purchased from Jia Cai Technology Co., Ltd. (Sichuan, China) are studied for DMA. The diameter, height and density of the material is 12 ± 2 mm, 12 ± 2 mm and 3–15 mg/cm^3^, respectively. Because the height of samples is supposed to be less than 10 mm for the DMA instrument under a compression pattern, the samples were cut in half by an ultraviolet picosecond laser precision machining system (Institute of Mechanics, Chinese Academy of Sciences). Finally, the height of the samples is reduced to be 6~8 mm and the diameter remains unchanged.

### 2.2. Experimental Instrument

The dynamic mechanical analysis (DMA) Q800 (Institute of Mechanics, Chinese Academy of Sciences) is made using a TA Instrument Inc. (109 Lukens Drive, New Castle, DE, USA) to study the viscoelastic properties of the GrFs as a function of temperature and loading frequency. Based on the size of the GrF samples, the compression clamp is used in the present work to fix the sample into the DMA instrument. When compared with the range of temperatures and loading frequencies in the previous studies [1,2,3,11], a larger temperature range of −50–500 °C and a frequency range of 0.1–30 Hz is be adopted and the effect of the oscillation amplitude on the viscoelastic behaviors of the GrF samples is explored using the experimental instrument. In addition, nitrogen is used to protect the sample from oxidation in the DMA experiments. When considering the height of the GrF samples and the loading frequency, the experimental strain rate is ~0.1 s^−1^.

### 2.3. Coarse-Grained Molecular Dynamics

**Figure 1:**
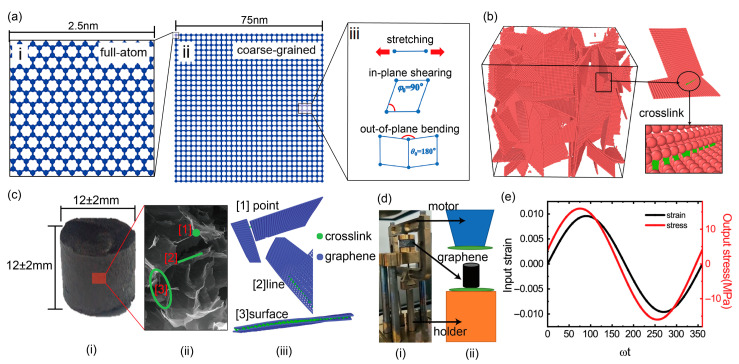
Coarse-grained model of GrFs and experiments of dynamic mechanical analysis. (**a-i**) A square full-atomic graphene sheet with a side length of 2.5 nm. (**a-ii**) A square coarse-grained graphene sheet with a side length of 75 nm. (**a-iii**) Three deformation modes of stretching, in-plane shearing and out-of-plane bending of graphene sheet. (**b**) The initial state of a well-equilibrated coarse-grained GrF consisting of 100 coarse-grained graphene sheets. The crosslinks (green) are added between neighbor beads in different sheets. (**c-i**) The experimental GrF, (**c-ii**) The SEM of GrF and (**c-iii**) the crosslinks between sheets. (**d-i**) The DMA process image and (**d-ii**) schematic image under compress pattern. (**e**) The cyclic strain and the stress as a function of the degree for the GrF sample at 300 K with a loading frequency of 500 MHz in simulation.

We use the coarse-grained graphene model given by Cranford et al. [14], which is proven effective in a series of studies about the mechanical deformations of a graphene nanoribbon [14] and graphene macro-assemblies [15,16]. In this method, a square graphene flake with a length of 2.5 nm and having 264 carbon atoms (Figure 1a-i) is reduced to a coarse grain. So, a larger square graphene flake with a length of 75 nm can be represented by 900 coarse beads connected by a set of linear springs and angular ones as shown in Figure 1a-ii,a-iii. The total energy of a graphene flake is calculated as:

$$E_{\mathrm{total}}=\sum_{i=1}^{N_{B}}E_{B}^{i}+\sum_{i=1}^{N_{\varphi }}E_{\varphi }^{i}+\sum_{i=1}^{N_{\theta }}E_{\theta }^{i}+\sum_{i=1}^{N_{\mathrm{LJ}}}E_{\mathrm{LJ}}^{i}$$

where *N*_B_, *N*_φ_, *N*_θ_ and *N*_LJ_ are the number of bonds (linear springs), in-plane angles, out-of-plane angles and bead pairs, respectively; $E_{B}^{i}$, $E_{\varphi }^{i}$, $E_{\theta }^{i}$ and $E_{\mathrm{LJ}}^{i}$ are the bond energy, in-plane angle energy, out-of-plane angle energy and van der Waals energy, respectively, and are calculated by Equations (1)–(4) in our simulations; the parameters of $k_{B}$, $k_{\varphi }$, $k_{\theta }$, $r_{0}$, $\varphi_{0}$, $\theta_{0}$, ε, σ and *r* are described in detail in our previous paper [18,19]. In practical GrF materials, a constituent graphene sheet contains about 1–10 graphene layers [4,20,21], in this paper, the GrF is composed of five layer sheets and is used to study the influence of temperature, frequency, oscillated amplitude, tensile and compressive pre-strain and density on the viscoelastic properties of GrF. Although graphene sheets in graphene foam materials are often imperfect, irregular in shape and have internal pores in real preparation processes, for simplicity, the graphene foam sample is composed of perfect graphene sheets, and the influence of the holes in the graphene sheets on the viscoelastic properties of the graphene foams is not considered. All the parameters for the coarse-grained 1–10 layers flakes are listed in the Table A1 at the end of this paper, which is obtained based on the equivalent energy principle by Cranford et al. [14].

(1) $$E_{B}=k_{B}{\left(r-r_{0}\right)}^{2}/2$$

(2) $$E_{\varphi }=k_{\varphi }{\left(\varphi -\varphi_{0}\right)}^{2}/2$$

(3) $$E_{\theta }=k_{\theta }{\left(\theta -\theta_{0}\right)}^{2}/2$$

(4) $$E_{\mathrm{LJ}}=4\varepsilon ({\left(\sigma /r\right)}^{12}-{\left(\sigma /r\right)}^{6})$$

Figure 1b shows the initial state of the GrF sample with 100 five-layer graphene sheets randomly distributed in the system as observed in the SEM experiment [20]. To mimic the connection between the neighboring sheets by physical crosslinks or functional groups [20,22], we use a crosslink model, which has been used to study the large-deformation and fracture of both the buckypapers [22,23] and the graphene foams [16,24]. The detailed parameters of the crosslink, numerical synthesizing method, energy minimization and the performed and visualized software are described in our previous paper [19]. As shown in Figure 1b, some crosslinks (green color) characterized by Equation (5) are added between the neighboring sheets in all the samples to enhance the connections. A macroscopic GrF sample and a local microstructure obtained by the SEM is shown in Figure 1c-i and c-ii, respectively. Three types of point, line and surface crosslinks are qualitatively described using our numerical model in Figure 1c-iii. The DMA in a compression pattern is shown in Figure 1d and the static force 0.1 N is applied to make the GrF stick to the device.

(5) $$E_{C}=k_{C}{\left(r-r_{0}\right)}^{2}/2$$

### 2.4. Dynamic Mechanical Analysis

The viscoelastic properties of the GrFs are analyzed numerically by a dynamic mechanical analysis (DMA), including the storage modulus, the loss modulus and the damping ratio. As a sinusoidal strain *ε* = *ε*_0_sin(*ωt*) is applied at one end of the equilibrated GrF sample in the x direction, a response of the stress *σ* = *σ*_0_ sin(*ωt* + *δ*) is activated as shown in Figure 1e, where *ε*_0_ and *σ*_0_ are the strain and the stress amplitude, respectively, *δ* is the phase angle, *ω* and *t* are the loading frequency and time. The storage and the loss modulus are *Y′* = *σ*_0_cos *δ*/*ε*_0_ and *Y″* = *σ*_0_sin*δ*/*ε*_0_, respectively. The damping ratio is calculated by tan *δ*. The loading amplitude is set to be a smaller value than 2 nm to ensure a linearly viscoelastic deformation. In the dynamic mechanical simulations, one atmosphere is maintained in the y and z direction as the dynamic loading is imposed in the x direction. Due to the limitation in the current computing power, the loading frequency in the CGMD simulations in this paper is in the range of 10^7^–10^9^ Hz, much higher than that in the DMA experiments. Considering the size of the numerical GrF samples and the high loading frequencies of up to 10^7^ Hz, the numerical strain rate is ~10^6^ s^−1^, much larger than that in the experiments. 

## 3. Results and Discussions

### 3.1. Effect of Density on Viscoelastic Properties of GrF

**Figure 2:**
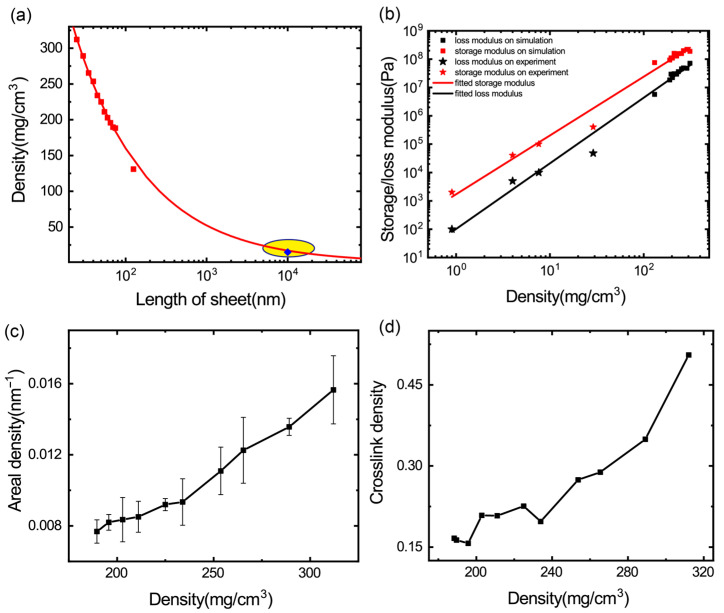
The density effect on viscoelastic properties of GrFs. (**a**) The relation between the density of GrFs and the length of constituent sheets (the red points are simulated data, the yellow circle signifies the range of experimental data from Jia Cai Technology Co., Ltd. (Sichuan, China). (**b**) The storage and loss modulus vary with the density of experimental samples (pentagram points) and numerical ones (square points). (**c**) The relation between the areal density (the contact area between all sheets divided by the volume of the system) and the density of numerical GrF samples. (**d**) The relation between the crosslink density (the number of inter-sheet crosslinks divided by the volume of the system) and the density of numerical GrF samples.

Density is the most important parameter to tune to the mechanical properties of porous materials. As shown in Figure 2a, we count the density and the size of the graphene sheets in our numerical samples (the red points) and the experimental samples (the yellow area) from Jia Cai Technology Co., Ltd. (Sichuan, China) and find that the density of the GrFs is nonlinearly dependent on the size of the constituent graphene sheets as shown in the red curve fitted using a power-law relationship *r/r*_0_= *a*(*l/l*_0_*)^n^*, where *r* is the density of the GrFs, *l* is the size of the graphene sheets and the two fitting parameters are *a* = 1496 ± 53.64, *n* = −0.49 ± 0.01. We further count the storage/loss modulus and the density of the GrFs in our simulations (square points) and those in the other experiments [2,3,25] (pentagram points) as shown in Figure 2b, and find that the storage/loss modulus is almost linearly dependent on the density as illustrated by the fitting curve *y* = *a* + *b* × *r*, the coefficients of the storage (*a*_S_ and *b*_S_) and the loss modulus (*a*_L_ and *b*_L_) are 3.23 ± 0.15, 2.07 ± 0.07, 2 ± 0.16 and 2.31 ± 0.07, respectively. The mechanism of the effect of density on the mechanical properties of the graphene foams was discussed in our previous work [24] and the work performed by Zhao Qin et al. [26]. For graphene foam to have a larger density, the connection points between the graphene sheets would have to be stronger because these connection points are always strengthened by the adhesion or crosslinks between the neighboring sheets. In this case, the deformation mode would be changed from the bending dominated to the stretching dominated, due to the in-plane stretching modulus of a paper-like graphene sheet (~1 TPa) that is much larger than the out-of-plane bending modulus, which can be used to qualitatively explain the huge increase in the storage modulus of the graphene foams as their density increases. In addition, according to our previous work [17] about the dissipation of graphene foams, all channels (rippling, sliding and impacting of the graphene sheets), the energy dissipation in the graphene foams is related to the connection between the graphene sheets, as the density of the graphene foam increases, The number of these microscale events of energy dissipation would undoubtedly increase, which can be used to qualitatively explain the increase in the loss modulus of the graphene foams.

To understand the linear dependency of the storage/loss modulus of the GrFs on density, we further examine the influence of density on the contact areal density calculated by the contact area between all the sheets divided by the system volume and crosslink density (the number of crosslinks between the sheets divided by the volume of the system). The areal density of the GrF is larger when the density of the GrF is larger as shown in Figure 2c. It is indicated that under the same amplitude and frequency, the GrFs of a higher density possess a larger sliding area; therefore, the loss modulus of the GrF increases as the density of the GrF increases. The crosslink density (the number of crosslinks between the sheets divided by the volume of the system) is larger when the density of the GrF is larger as shown in Figure 2d. The crosslinks enhance the connections between the sheets and it is not easy to slide apart these sheets, therefore the storage modulus of GrF increases as the density of the GrF increases.

### 3.2. Effect of Temperature 

**Figure 3:**
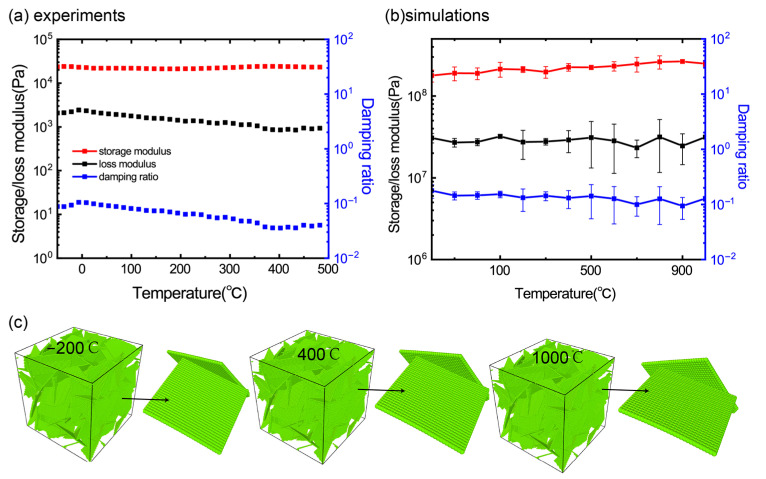
The effect of temperature on the viscoelastic properties of (**a**) the experimental GrF sample at a given low frequency in a temperature range and (**b**) the numerical sample at a given high frequency in a temperature range. (**c**) Similar microstructures and a local edge-surface contact in numerical samples of GrFs at different temperatures of −200, 400 and 1000 °C.

Figure 3a shows the effect of temperature, in the range of −50 to 500 °C, has on the storage/loss modulus and the damping ratio. It is found that all three viscoelastic quantities are almost insensitive to the temperature, showing a temperature-independent viscoelastic characteristic. Here, we noted that the loss modulus decreases slightly as the temperature increases and the change is within an order of magnitude. As we only performed a group of experiments and cannot give the mean and variance of the loss modulus, it is not clear whether the slight reduction in the loss modulus is the actual response of the material or the experimental fluctuation. However, when compared with other viscoelastic materials (e.g., rubber), which is highly sensitive to temperature, the viscoelastic behavior that are almost independent on temperature are still the most distinctive characteristics of graphene foam materials. A similar phenomenon was also observed in the experiments [1,3]. Furthermore, in order to break through the limitation of the experimental instruments on the temperature range, we adopted the CGMD simulations to study the effect of the viscoelastic properties of the GrFs using a wider temperature range from −200 to 1000 °C. As shown in Figure 3b, the storage/loss modulus and the damping ratio of the numerical GrF samples exhibit a similar temperature-independent characteristic as that observed in our experiment and others. Here, we note that another carbon-based material of the buckypaper, which is an assembly of a large number of carbon nanotubes that has the same temperature-independent viscoelastic properties. The underlying mechanism is ascribed to the good thermostability of carbon materials and the existence of inter-sheet adhesion and crosslinking, limiting the aggregation and crimping behavior of the thick graphene sheets. Due to this reason, the structures of the GrFs under cyclic loadings are almost unchanged at varied temperatures of −200, 400 and 1000 °C as shown in Figure 3c. It is much different from the traditional temperature-dependent polymer materials [27,28] and polymer-based composites [9,29].

Although both experimental samples and numerical ones have the same temperature-insensitive viscoelastic properties, the storage/loss modulus of the experimental samples is much smaller than that of the numerical samples. The great difference of the storage/loss modulus is mainly induced by the density of the samples as discussed in Figure 2b. The density of the experimental samples is about 10 mg/cm^3^, almost an order of magnitude smaller than that of numerical ones (about 100 mg/cm^3^). Due to the restriction of the computation condition, it is difficult for us to construct a numerical sample of graphene foams with a small density and similar microstructures as that of the experimental samples. This is because the density of the graphene foam is highly related to the size of the graphene sheets, for a numerical graphene foam with a small density ~10 mg/cm^3^ as that in the experiments, the size of the constituent graphene sheets should be as large as ~1 micrometer. If the coarse-grained scheme of the graphene sheet is adopted, the system will contain 16 million coarse grains, which is beyond the computation ability of my group.

### 3.3. Effect of Frequency

**Figure 4:**
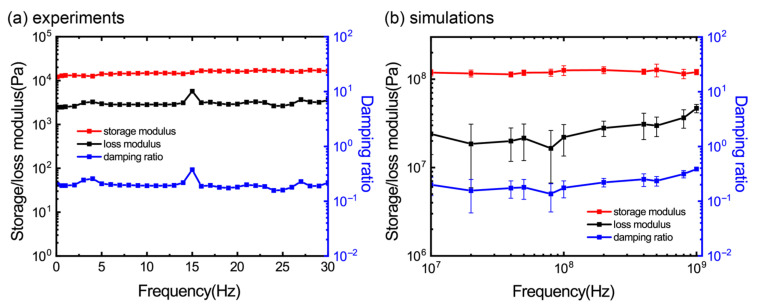
The effect of frequency on viscoelastic properties of GrFs by (**a**) experiments and (**b**) simulations.

To study the effect of a wider loading frequency on the viscoelastic properties of the GrFs, we combine both the experimental and numerical DMA to apply cyclic loadings with both a low and high frequency to our experimental/numerical samples, respectively. Figure 4a shows the storage modulus, loss modulus and the damping ratio of the experimental samples as a function of the frequency in a broad range from 0.1 Hz to 30 Hz, which is larger than that reported in the references [1,3]. There is a small peak at 15 Hz in the curve of the loss modulus, which may be a larger experimental fluctuation because we did not observe any differences in the experiment process as the frequency is set to 15Hz. It can be seen that the three quantities are almost unchanged in the whole frequency spectrum. Interestingly, the similar frequency-insensitive characteristics of the viscoelastic properties of the GrFs can be reproduced in our numerical samples in the DMA simulations at a high loading frequency from 10^7^ to 10^9^ Hz as shown in Figure 4b. As discussed in Figure 3, the density of our numerical samples is ~100 mg/cm^3^,which is an order of magnitude larger than that of the experimental samples ~10 mg/cm^3^. This difference in density should be responsible for the huge increase in the storage/loss modulus of the numerical samples when compared to the experimental ones as discussed in Figure 2b.

### 3.4. Effect of Loading Amplitude

**Figure 5:**
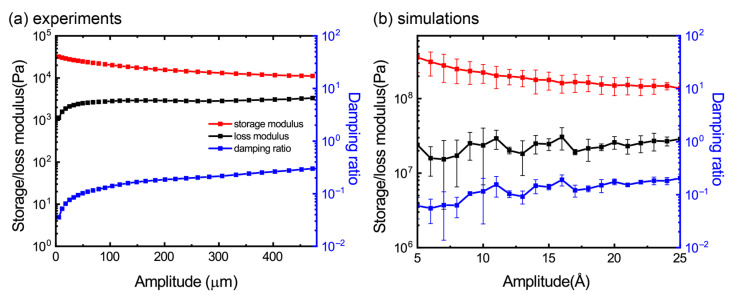
The amplitude effect on viscoelastic properties of GrF on (**a**) experiments and (**b**) simulations.

Figure 5 shows the effect of the strain amplitude, i.e., the quantity *ε*_0_ in the strain load *ε* = *ε*_0_sin(*ωt*) on the storage modulus, loss modulus and the damping ratio of the GrF materials by using both the experimental and numerical DMA. In Figure 5a, the storage modulus of the experimental samples decreases constantly with an increased loading amplitude, while the loss modulus increases as the amplitude is smaller than ~50 μm and keeps nearly constant. The variation in the two moduli is within an order of magnitude. Interestingly, the similar characteristics can be observed in the corresponding numerical graph as shown in Figure 5b. These phenomena can be well explained using our previous studies on the elasticity [24] and energy dissipation [29] of the GrFs under uniaxial tension and compression According to our studies, the elasticity of GrFs would be weakened with an increased tensile strain due to bond breaking and some irreversible reconstitution of the microstructures, while the dissipation ability would be strengthened due to the enhanced ripping, sliding and impacting of the graphene sheets under a cyclic loading with a larger strain. However, if the loading magnitude is too large, the phenomenon of the deformation localization would emerge, which would in turn deteriorate the ability of energy dissipation.

### 3.5. Effect of Compressive and Tensile Pre-Strain

**Figure 6:**
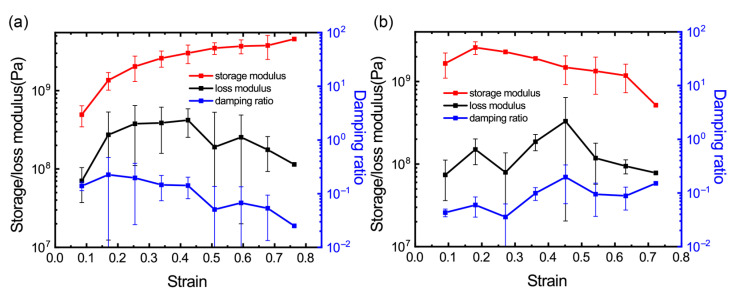
The effect of compressive and tensile strain on the viscoelasticity of GrFs. (**a**) The storage and loss modulus and the damping ratio vary with compressive pre-strain. (**b**) The storage and loss modulus and the damping ratio vary with tensile pre-strain.

In order to demonstrate the dependency of viscoelasticity of the GrFs on their deformations, we apply a compressive and tensile strain before conducting the numerical DMA simulations. The graphene foam sample, as given in Figure 1b, was first compressed or stretched to a certain strain, and then, the deformed sample is chosen to conduct the DMA simulations to study the viscoelastic response of the graphene foam sample at a certain pre-strain. We, in total, chose nine intermediate configurations with varied compressive strains and eight intermediate configurations with varied tensile strains for the following numerical dynamic mechanical analysis. As shown in Figure 6a, the storage modulus increases constantly with the increased compressive strain. This phenomenon can be well explained by our previous work [24] on the elasticity of the GrFs under compression. As the compressive strain increases, more graphene sheets are bended and more bended sheets experience larger bending deformations. These bended graphene sheets, especially those laminated thick sheets, act as storage units and contribute to the increased modulus. The loss modulus, however, is almost unchanged considering the large standard deviations. We also study the viscoelasticity of the GrFs after a tensile strain, as shown in Figure 6b, the storage modulus is decreased due to some bonds between the neighboring graphene sheets breaking as the tensile strain increases. Considering the large standard deviations, the loss modulus is almost unchanged.

## 4. Conclusions

In this paper, a combination of the DMA experiments and the CGMD simulations is adopted to study the viscoelastic behaviors of GrF materials. The effect of density of the GrFs, temperature, loading frequency, oscillation amplitude, pre-strain on the storage and loss modulus as well as the damping ratio of the GrFs are considered. We first give the density dependence of the storage/loss modulus of the GrFs using the data from the present work and other references, and it is found that the storage/loss modulus is almost linearly dependent on the density as illustrated by the fitting curve *y* = *a* + *b* × *r*, the coefficients of the storage and loss modulus are 3.23 ± 0.15, 2.07 ± 0.07, 2 ± 0.16 and 2.31 ± 0.07, respectively. Furthermore, we explain the physical mechanism of the effect of density, that is, as the density of the graphene foams increases the storage modulus increases greatly because the deformation mode of more constituent sheets changes from the bending dominated to the stretching dominated; the loss modulus increases because more contact points between the graphene sheets participate in the energy dissipation process. Then, benefiting of the advantages of both the experiment and CGMD method, we study the viscoelastic behaviors of the GrFs with a larger range of GrF densities, temperatures (from −20 to 1000 °C) and loading frequencies (0–30 Hz in the experiments and 10^7^–10^9^ Hz in the CGMD). Although the value of the storage/loss modulus varies greatly from about 10^7^–10^4^ Pa in the CGMD simulations and the DMA experiments due to the variation in the GrF density and the inter-sheet crosslinks, the temperature- and frequency-independent viscoelastic behaviors of the GrFs are observed both in our CGMD simulations and in the experiments. For the effect of strain amplitude, it can be observed in both the CGMD simulations and experiments that the storage modulus decreases slightly as an increasing strain amplitude due to the bond-breaking, and the loss modulus increases slightly when the strain amplitude is relatively small and then saturates. Furthermore, the tensile/compressive pre-strain can be used to effectively tune the storage modulus of the GrFs. The storage modulus increases constantly with an increased compressive strain while it decreases due to some bonds between the neighboring sheets breaking as the tensile strain increases. The loss modulus is almost unchanged in both cases. These results would be helpful not only for understanding the mechanical mechanism of GrFs but also for the design of GrFs with advanced properties.

## Data Availability

Data sharing is not applicable.

## Appendix A

**Table A1:** The parameters of the main force field.

Parameters	No. of Graphene Layers										Units
	1	2	3	4	5	6	7	8	9	10	
*k*_T_	470	930	1396	1860	2323	2787	3253	3720	4190	4663	kcal mol^−1^Å^−2^
*k*_φ_	16,870	33,740	50,610	67,480	84,350	101,220	118,090	134,960	151,829	168,698	kcal mol^−1^rad^−2^
*σ*	2.98	5.96	8.78	11.92	15.20	18.35	21.27	23.84	25.89	27.30	Å
*k*_θ_	144.9	8970	28,782	82,731	185,601	351,166	595,220	933,087	1,379,947	1,951,198	kcal mol^−1^rad^−2^

## References

[1] Luo Y., Jiang S., Xiao Q., Chen C., Li B. (2017). Highly reusable and superhydrophobic spongy graphene aerogels for efficient oil/water separation. Sci. Rep..

[2] Sun H., Xu Z., Gao C. (2013). Multifunctional, ultra-flyweight, synergistically assembled carbon aerogels. Adv. Mater..

[3] Wu Y., Yi N., Huang L., Zhang T., Fang S., Chang H., Li N., Oh J., Lee J.A., Kozlov M. (2015). Three-dimensionally bonded spongy graphene material with super compressive elasticity and near-zero Poisson’s ratio. Nat. Commun..

[4] Chen Z., Ren W., Gao L., Liu B., Pei S., Cheng H.-M. (2011). Three-dimensional flexible and conductive interconnected graphene networks grown by chemical vapour deposition. Nat. Mater..

[5] Ma Y., Chang H., Zhang M., Chen Y. (2015). Graphene-based materials for lithium-ion hybrid supercapacitors. Adv. Mater..

[6] Yavari F., Chen Z., Thomas A.V., Ren W., Cheng H., Koratkar N. (2011). High sensitivity gas detection using a macroscopic three-dimensional graphene foam network. Sci. Rep..

[7] Wang X., Lu M., Wang H., Pei Y., Rao H., Du X. (2015). Three-dimensional graphene aerogels-mesoporous silica frameworks for superior adsorption capability of phenols. Sep. Purif. Technol..

[8] Chen S., Bao P., Huang X., Sun B., Wang G. (2014). Hierarchical 3D mesoporous silicon @ graphene nanoarchitectures for lithium ion batteries with superior performance. Nano Res..

[9] Sadasivuni K.K., Ponnamma D., Kumar B., Strankowski M., Cardinaels R., Moldenaers P., Thomas S., Grohens Y. (2014). Dielectric properties of modified graphene oxide filled polyurethane nanocomposites and its correlation with rheology. Compos. Sci. Technol..

[10] Boland C.S., Khan U., Ryan G., Barwich S., Charifou R., Harvey A., Backes C., Li Z., Ferreira M.S., Möbius M.E. (2016). Sensitive electromechanical sensors using viscoelastic graphene-polymer nanocomposites. Science.

[11] Xu Y., Sheng K., Li C., Shi G. (2010). Self-assembled graphene hydrogel via a one-step hydrothermal process. ACS Nano.

[12] Qiu L., Liu J., Chang S., Wu Y., Li D. (2012). Biomimetic superelastic graphene-based cellular monoliths. Nat. Commun..

[13] Shen Z., Ye H., Zhou C., Kröger M., Li Y. (2018). Size of graphene sheets determines the structural and mechanical properties of 3D graphene foams. Nanotechnology.

[14] Cranford S., Buehler M. (2011). Twisted and coiled ultralong multilayer graphene ribbons. Model. Simul. Mater. Sci. Eng..

[15] Wang C., Zhang C., Chen S. (2016). The microscopic deformation mechanism of 3D graphene foam materials under uniaxial compression. Carbon.

[16] Pan D., Wang C., Wang T., Yao Y. (2017). Graphene foam: Uniaxial tension behavior and fracture mode based on a mesoscopic model. ACS Nano.

[17] Wang C., Pan D., Chen S. (2018). Energy dissipative mechanism of graphene foam materials. Carbon.

[18] Wang C., Chen S. (2016). Viscoelastic properties of randomly entangled carbon nanotube networks under cyclic tension loading. Comput. Mater. Sci..

[19] Liu S., Lyu M., Wang C. (2021). Mechanical properties and deformation mechanisms of graphene foams with bi-Modal sheet thickness by coarse-grained molecular dynamics simulations. Materials.

[20] Nieto A., Boesl B., Agarwal A. (2015). Multi-scale intrinsic deformation mechanisms of 3D graphene foam. Carbon.

[21] Bi H., Chen I., Lin T., Huang F. (2015). A new tubular graphene form of a tetrahedrally connected cellular structure. Adv. Mater..

[22] Yang T., Wang C., Wu Z. (2020). Crosslink-tuned large-deformation behavior and fracture mode in buckypapers. Carbon.

[23] Wang C., Wang L., Xu Z. (2013). Enhanced mechanical properties of carbon nanotube networks by mobile and discrete binders. Carbon.

[24] Wang C., Zhang C., Chen S. (2019). Micro-mechanism and influencing factors of graphene foam elasticity. Carbon.

[25] Zhao W., Elias A.L., Rajukumar L.P., Kim H.-I., O’Brien D.J., Zimmerman B.K., Penev E.S., Terrones M., Yakobson B.I., Wei B. (2015). Controllable and predictable viscoelastic behavior of 3D boron-doped multiwalled carbon nanotube sponges. Part. Part. Syst. Charact..

[26] Zhao Q., Jung G., Kang M., Buehler M. (2017). The mechanics and design of a lightweight three-dimensional graphene assembly. Sci. Adv..

[27] Lakes R.S. (2009). Viscoelastic Materials.

[28] Zeng X., Ye L., Sun R., Xu J., Wong C. (2015). Observation of viscoelasticity in boron nitride nanosheet aerogel. Phys. Chem. Chem. Phys..

[29] Zhang H.B., Zheng W., Yan Q., Jiang Z., Yu Z. (2012). The effect of surface chemistry of graphene on rheological and electrical properties of polymethylmethacrylate composites. Carbon.

